# Supplementary material for: History is written by the victors: The effect of the push of the past on the fossil record
Source: Evolution. 2018 Sep 26;72(11):2276–91. doi: 10.1111/evo.13593 (PMC6282550; doi:10.1111/evo.13593)
Supplement: Supplementary file 1 — run_experiments_SI.R gives the R commands necessary to produce each of the figures in the manuscript (excluding figure 3). fossil_trees_SI.R contains the source code necessary to perform all the mathematical analyses described in the manuscript, and should be ‘sourced’ prior to running the commands in run_experiments_SI.R [file EVO-72-2276-s001.zip › README.rtf]

Supporting Information for Mann & Budd: History is written by the victors: the effect of the push of the past on the fossil recordrun_experiments_SI.R gives the R commands necessary to produce each of the figures in the manuscript (excluding figure 3).fossil_trees_SI.R contains the source code necessary to perform all the mathematical analyses described in the manuscript, and should be ‘sourced’ prior to running the commands in run_experiments_SI.R
